# *Akkermansia muciniphila* Suppresses High-Fat Diet-Induced Obesity and Related Metabolic Disorders in Beagles

**DOI:** 10.3390/molecules27186074

**Published:** 2022-09-17

**Authors:** Xiao-Qi Lin, Wei Chen, Ke Ma, Zhen-Zhen Liu, Yu Gao, Jian-Gang Zhang, Tao Wang, Yong-Jun Yang

**Affiliations:** Key Laboratory of Zoonosis Research, Ministry of Education, College of Veterinary Medicine, Jilin University, Changchun 130062, China

**Keywords:** *Akkermansia muciniphila*, obesity, gut microbiota, beagle dog, microcapsule

## Abstract

Obesity is one of the prevalent chronic diseases in human and companion animals usually associated with several metabolic disorders. The gut commensal bacterium *Akkermansia muciniphila* (*A. muciniphila*) is known for its therapeutic effects on metabolic disorders and inflammations. Here, we isolated the *A. muciniphila* AKK2 strain from the feces of interferon-inducible protein 204^−/−^ (IFI204^−/−^) mice and further evaluated its anti-obesity effects on high-fat diet (HFD)-fed C57BL/6J mice and beagles. The results showed that it effectively controlled weight gain. Microbiome analysis using 16S rRNA gene sequencing revealed that HFD alters gut microbiota composition and *A. muciniphila* AKK2 increases the *Firmicutes/Bacteroidetes* (F/B) ratio in beagles. Furthermore, we prepared microcapsules containing *A. muciniphila* AKK2, and tolerance tests showed the encapsulation maintained high viability and stability in an aerobic environment and simulated the secretion of gastrointestinal fluids. Overall, this study widens the spectrum of *A. muciniphila* applications to prevent obesity.

## 1. Introduction

Obesity is the excessive accumulation of body fat, mainly due to genetics, environmental factors, and the gut microbiota. Increasing evidence shows that long-term obesity may trigger specific metabolic disorders, like cardiovascular diseases, dyslipidemia, asthma, insulin resistance, type 2 diabetes, non-alcoholic fatty liver disease (NAFLD) and some types of cancer [1,2,3,4]. It has been reported that about 1.1 billion adults are overweight and 312 million are obese [5]. Moreover, it has been reported that about 60% of dogs and cats are overweight or obese [6,7]. Therefore, its prevention and treatment in humans and companion animals is a serious societal concern [8,9].

In recent years, probiotics has been used to treat obesity [10]. Specific bacteria are associated with obesity and related metabolism, so they can be regarded as therapeutic targets. As such, the intestinal mucin-degrading symbiont *Akkermansia muciniphila* is generally considered to be important for maintaining gut health and glucose homoeostasis [11]. However, effective probiotic therapies with anti-obesity applications for cats and dogs are not available [12], perhaps because of the diverse gut microbiota of these species. Consequently, typical therapies to treat obese pets are changes in exercise and diet [13].

Recent studies have found that the relative abundance of *A. muciniphila* decreases in obese mice, whereas administration of fructooligosaccharides to obese mice increases it approximately 100-fold [14]. Administration of live *A. muciniphila* reverses high-fat diet-induced metabolic disorders, which might be associated with an increase in the intestinal levels of endocannabinoids [15]. However, its anti-obesity effect on dogs has yet to be revealed, and the exact physiological function it plays during these processes has remained elusive.

To verify its anti-obesity function and provide a rationale for its sustainable use and commercial development in the treatment of dogs, we isolated *A. muciniphila* strains from mice and investigated anti-obesity in mice and dogs.

## 2. Results

### 2.1. Isolation, Identification and Selection of Bacterial Strains

**Figure 1:**
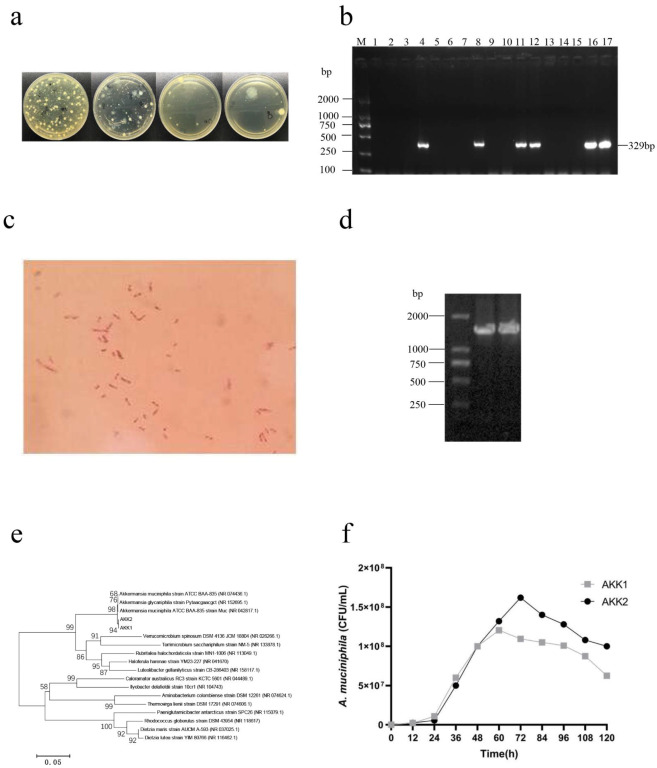
*A. muciniphila* strains were isolated from mouse feces. (**a**) The colony morphology on vancomycin solid medium. (**b**) The PCR gel electrophoresis of suspected *A. muciniphila* strains. M: DL2000 DNA Marker; 1–17: suspected isolates. (**c**) Gram staining of strains (1000×). (**d**) PCR products of isolates AKK1 and AKK2 on agarose gel. M, DL2000 DNA Marker; 1–2: Suspected isolates. (**e**) Phylogenetic analysis of strains by maximum likelihood method and the general time reversible model. (**f**) growth curve of *A. muciniphila* AKK1 and AKK2.

In our previous studies, we observed that the abundance of *A. muciniphila* in IFI204-deficient mice (C57BL/6 background) was higher than in C57BL/6 control mice. Thus, *A. muciniphila* was isolated from freshly voided fecal samples of IFI204-deficient mice using vancomycin agar plates. The colonies appeared after 7 days of anaerobic culturing at 37 °C (Figure 1a). These isolates were further subcultivated and purified on BHI plates. The purified colonies were off-white translucent, with a smooth surface, tiny round shape, and neat edges. The suspected colonies were identified by PCR. Electrophoresis results showed that the bands of 6 strains appeared at about 329 bp (Figure 1b). Gram staining and a 100-fold oil microscope showed that the suspected strains were red short-rod shaped, which indicated Gram-negative bacteria (Figure 1c). The 16S rRNA gene amplification results of isolated bacteria revealed two strains to be positive (Figure 1d). Evolutionary tree analysis showed that the isolates AKK1 and AKK2 had 99% similarity to *A. muciniphila* BAA-835 (Figure 1e). To select a better strain for subsequent experiments, we evaluated the growth characteristics of two AKK1 and AKK2. The number of viable bacteria in the log phase of AKK2 was more than that of AKK1, which was 1.6 × 10^8^ CFU/mL (Figure 1f). The strains were further tested for resistance to low pH and bile, important characteristics for surviving transit through the gastrointestinal tract [16]. For this purpose, an agar-based screening protocol was performed, with results showing that AKK1 and AKK2 had different survival rates under different pH and bile salt concentration conditions (Table 1, Table 2 and Table 3). It is well known that under physiological conditions, the average concentration of bile salts is believed to be 0.3% (*w*/*v*) in the intestines [17,18,19]. There was no significant difference in the survival at this bile salt concentration. However, the survival rate of AKK2 was better than for AAK1 under different pH values. Thus, AKK2 was selected for the following experiment.

### 2.2. Effects of A. muciniphila on the Body Weight and BCS in the HFD-Fed Animals

**Figure 2:**
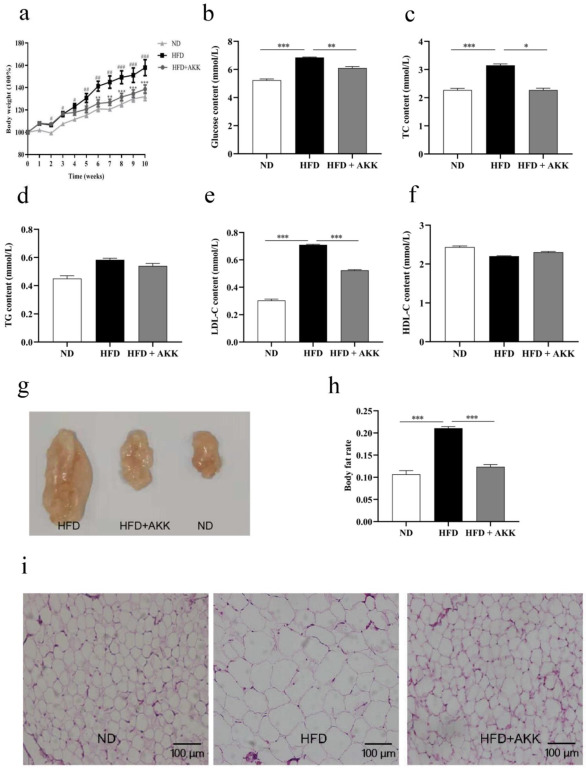
Effects of pasteurized *A. muciniphila* strain AKK2 on HFD-fed obese mice. (**a**) body weight measured weekly (data expressed as % of initial body weight). (**b**) serum level of fasting glucose, (**c**) total cholesterol (TC), (**d**) total triglyceride (TG), (**e**) LDL cholesterol (LDL-C) and (**f**) HDL cholesterol (HDL-C) in different experimental groups. (**g**) total abdominal fat, (**h**) body fat rate. (**i**) inguinal fat HE staining in different groups. The data are presented as the mean ± SEM (*n* = 8). As for body fat rate, data expressed as the weight of total abdominal fat/body weight. Statistical analyses were performed with one-way ANOVA test. * *p* < 0.05, ** *p* < 0.01 and *** *p* < 0.001.

**Figure 3:**
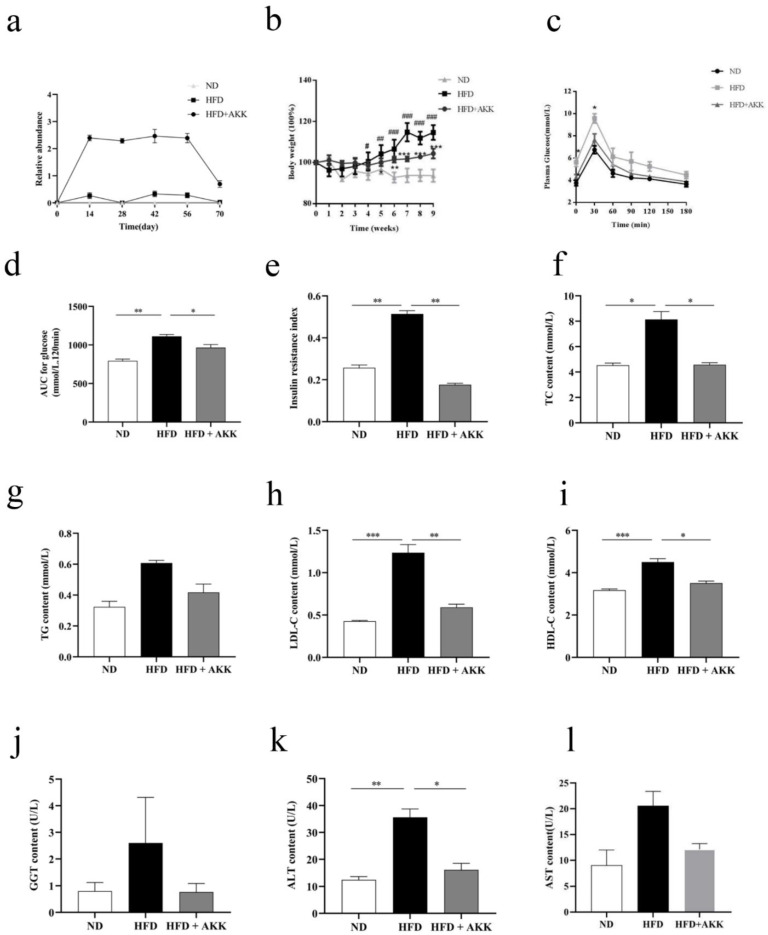
*A. muciniphila* AKK2 reduced body weight and improved metabolism in HFD-induced beagles. (**a**) relative abundance of *A. muciniphila* in dog gastrointestinal tract. (**b**) body weight measured weekly (data expressed as % of initial body weight). (**c**) serum glucose levels at different time-points and (**d**) area under the curve (AOC) in oral glucose tolerance tests (OGTT). (**e**) insulin resistance index. (**f**) serum level of total cholesterol (TC). (**g**) triglyceride (TG). (**h**) LDL cholesterol (LDL-C). (**i**) HDL cholesterol (HDL-C). (**j**) transglutaminase (GGT). (**k**) alanine aminotransferase (ALT). (**l**) aspartate aminotransferase (AST) for different experimental groups. The data are presented as the mean ± SEM (*n* = 8). Statistical analyses were performed with one-way ANOVA test. * *p* < 0.05, ** *p* < 0.01 and *** *p* < 0.001.

Several lines of evidence have shown that *A. muciniphila* inhibits obesity and related metabolic disorder in mice [15,20,21,22]. Therefore, we evaluated whether pasteurized AKK2 strain supplements had an effect in HFD-induced obese mice. The results showed they decreased body weight gain (Figure 2a), fat body rate, fasting blood glucose, serum TC and LDL-C levels significantly (Figure 2b–h), and they improved fat vacuoles in the HFD-fed mice (Figure 2i), which indicated that they also had a protective effect on HFD-induced obese mice. To determine the effect of *A. muciniphila* strain AKK2 on body weight in beagles, we established an obese canine model by feeding beagles a high-fat diet. Detection of *A. muciniphila* in feces suggested successful gastrointestinal transit following oral administration (Figure 3a). The results showed that treatment of the HFD-fed beagles with AKK2 reduced weight gain significantly (Figure 3b).

At week 9, the BCS of the beagles in the ND and HFD + AKK2 groups did not change significantly compared with what it was before dietary intervention, whereas, the BCS of beagles in the HFD group was 8.95 ± 0.51, which was significantly higher than before dietary intervention (Table 4). The above results indicated *A. muciniphila* AKK2 had a protective effect on HFD-fed obese beagles.

### 2.3. A. muciniphila Ameliorated Glucose Homeostasis, Blood Lipid Accumulation and Liver Damage in HFD-Induced Beagles

Next, we determined the effect of *A. muciniphila* on high-fat diet-induced blood glucose disorders. The results of the OGTT and ITT showed that *A. muciniphila* alleviated the fasting serum glucose and insulin resistance level significantly (Figure 3c–e).

Previous studies revealed some metabolic features of obesity, such as high serum total cholesterol (TC), high TG [23], low HDL-C, and several metabolic diseases [24]. To determine whether *A. muciniphila* improves abnormalities in serum metabolic parameters in obese beagles, the concentrations of serum TC, total TG, HDL-C, and LDL-C were analyzed (Figure 3f–i). As expected, the results showed that *A. muciniphila* decreased these obesity parameters significantly, though the TG level was not significant. All of these results indicated that *A. muciniphila* significantly improved blood metabolic parameters in obese beagles on a high-fat diet.

ALT and AST are commonly used to assess liver functions or liver injury. Thus, we measured serum AST, GGT, and ALT levels to determine the effect of *A. muciniphila* on the liver. We found increased level of ALT in mice fed a high-fat diet, although AST and GGT were not significant (Figure 3j–l). Nevertheless, supplements of *A. muciniphila* AKK2 depleted these parameters significantly to their normal levels in the HFD-fed beagles, indicating AKK2 could alleviate liver dysfunction.

### 2.4. The Regulating Effects of A. muciniphila on the Gut Microbiota in the HFD-Induced Obese Beagles

**Figure 4:**
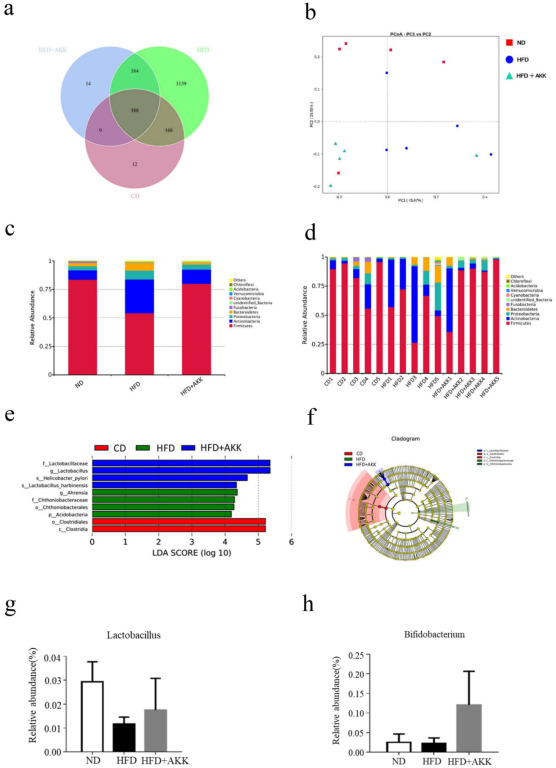
*A. muciniphila* modulated the gut microbiota in HFD-fed beagles. (**a**) Venn diagrams of the gut microbiota, (**b**) principal co-ordinates analysis, (**c**) microbial composition at the phylum level (top 10) of different groups, (**d**) microbial composition at the phylum level (top 10) of different samples, (**e**) linear discriminant analysis (LDA) score (log 10), (**f**) cladogram, (**g**,**h**) abundance of *Lactobacillus* and *Bifidobacterium*, respectively.

The analysis of the fecal flora indicated that the OTU numbers decreased after treatment with *A. muciniphila* AKK2 (Figure 4a). The differences between fecal microbial samples from each group were further analyzed by principal co-ordinate analysis (PCoA). The results demonstrated unique microbial clustering in different groups although some samples from the HFD + AKK group overlapped with those of the HFD group (Figure 4b).

**Figure 5:**
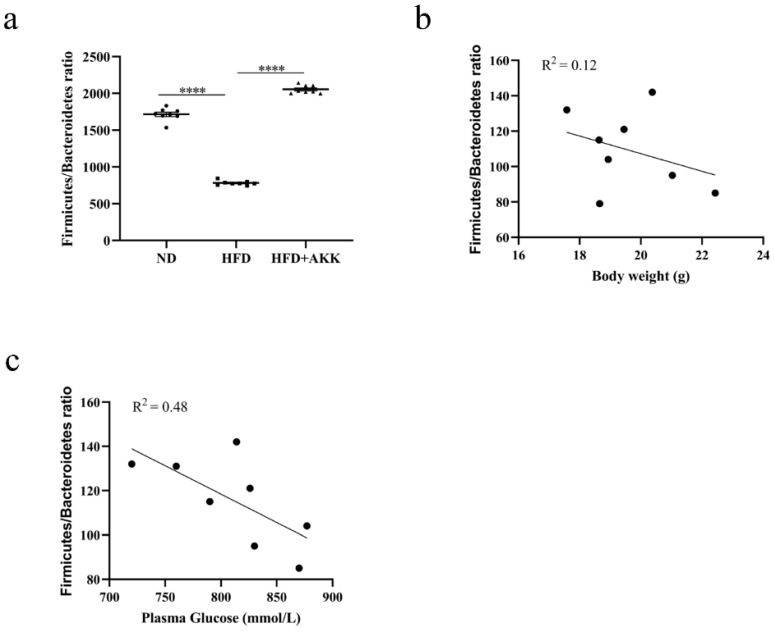
Correlation analysis of gut microbiota and obesity phenotype in HFD-induced beagles. (**a**) The *Firmicutes/Bacteroidetes* ratio in different groups. (**b**) Correlation between body weight and the *Firmicutes/Bacteroidetes* ratio (Correlation coefficient R^2^ = 0.12). (**c**) Correlation between plasma glucose and the *Firmicutes/Bacteroidetes* ratio (Correlation coefficient R^2^ = 0.48). Statistical analyses were performed with unpaired Student-*t* test. **** *p* < 0.0001.

At the phylum level, the dominant bacteria were *Bacteroidetes*, *Actinobacteria*, and *Firmicutes*, accounting for more than 80% of the intestinal flora. However, HFD feeding decreased the abundance of *Firmicutes* significantly but increased *Bacteroidetes*, *Actinobacteria*, and Proteobacteria markedly. However, *A. muciniphila* AKK2 modulated these changes significantly in the HFD-fed beagles. Therefore, we speculated that *A. muciniphila* AKK2 restored the imbalance of intestinal flora caused by a high-fat diet (Figure 4c,d). Compared with the HFD group, some taxa, especially *Lactobacillus*, were significantly enriched in the HFD + AKK group (LDA > 5.0) (Figure 4e,f). Furthermore, at the genus level, the relative abundance of *Bifidobacterium* and *Lactobacillus* increased after supplementation with *A. muciniphila* AKK2 compared to the HFD group although the difference was not significant (Figure 4g,h). Correlations between body weight and *Firmicutes/Bacteroidetes* with or without blood glucose were determined by calculating Pearson’s correlation coefficient [25] (Figure 5a–c).

### 2.5. Preparation, Stability, in Vitro Simulation of Gastrointestinal Digestion of Microcapsules

Previous studies reported that alginate hydrogel was an ideal material for bacteria encapsulation [26,27]. Here, *A. muciniphila* AKK2 was mixed with alginate, and droplets were formed using a 1 mL injector. To improve retention efficiency, chitosan with calcium was added into the collection tube to allow for simultaneous coating and crosslinking of the alginate droplets.

**Figure 6:**
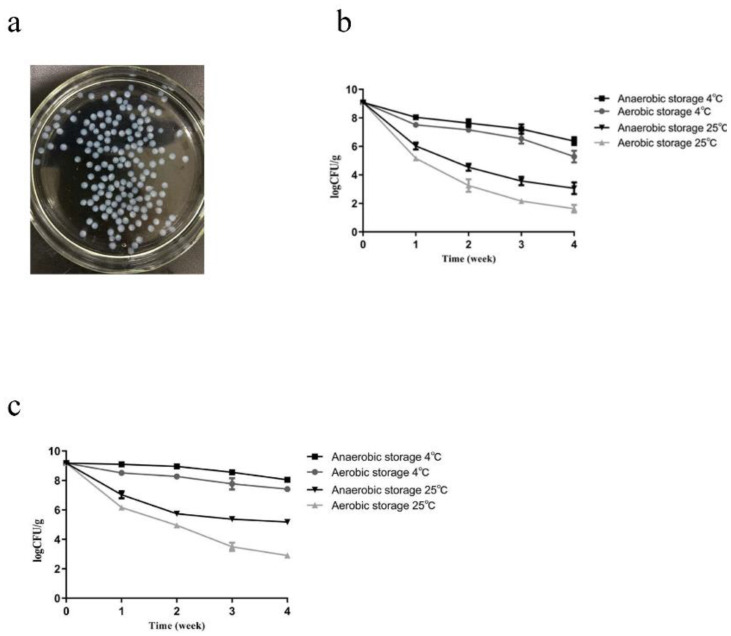
The morphology and characteristics of *A. muciniphila* strain AKK2 microcapsule. (**a**) morphology of alginate/chitosan encapsulated AKK2 microcapsule. (**b**) preserving stability of unencapsulated AKK2 and (**c**) encapsulated AKK2 at different time points.

The size and shape of the microcapsules were uniform, spherical, smooth, and round, with an average diameter of 1.86 ± 0.28 mm (Figure 6a). The embedding rate was 81.8%, so sodium alginate, pectin, and chitosan as wall materials have a good embedding effect (Table 5).

The values are presented as the mean ± SEM. Embedding rate = number of viable bacteria before embedding minus the number of viable bacteria in the filtrate and washing liquid)/number of viable bacteria before embedding × 100.

The storage stability tests of AKK2 and AKK2 microcapsules showed that they both exhibited high stability and survival under 4 ℃ anaerobic conditions. Moreover, under testing conditions, the survival rate of AKK2 microcapsules was significantly higher than that of the lyophilized powder of unencapsulated bacteria (Figure 6b,c), which indicated that probiotic microencapsulation improved stability and the number of viable bacteria during processing and storage.

The microcapsules were tested for the release of live bacteria after simulated gastric incubation. The *A. muciniphila* AKK2 release rate from the microcapsules when incubated in simulated gastric fluid (SGF) followed by simulated intestinal fluid (SIF) was tested (Table 6). The counts of live AKK2 decreased by 4.51 log (CFU/g) compared to the initial viable cell count, but AKK2 microcapsules decreased by only 1.99 log (CFU/g). Compared to the initial viable cell count, the decrease was significantly lower than that of nonencapsulated bacteria. These results suggested that microencapsulated AKK2 exhibited excellent viability and stability in SGF and SIF.

## 3. Materials and Methods

### 3.1. Isolation, Identification and Selection of A. muciniphila

The fresh fecal samples of interferon-inducible protein 204 (IFI204)-deficient male mice were collected and suspended in a sterile synthetic liquid medium (3.8% *w*/*v* Brain Heart Infusion (BHI) powder, 1.6% *w*/*v* soya peptone, 0.4% *w*/*v* threonine, 25 mM glucose, 25 mM N-acetylglucosamine, pH 7.2–7.4) and homogenized for 3 min. One hundred microliter aliquots were taken and serially diluted 10-fold with sterile physiological saline. Then, 0.1 mL of appropriate dilutions was spread onto BHI agar supplemented with 4 g/L mucin and 5 μg/mL vancomycin (Vancomycin agar), which were then incubated at 37 °C under anaerobic conditions (85% N_2_, 10% CO_2_ and 5% H_2_) for 5–7 d. Colonies with different morphologies were picked out and subcultured onto fresh vancomycin agar plates until the cultures were deemed to be pure. Isolated colonies were characterized preliminarily on the basis of morphology and Gram staining. Furthermore, each isolate was PCR-amplified using the *A. muciniphila* specific primers (forward, 5-GCATATCAATAAGCGGAGGAAAAG; reverse, 5-GGTCCGTGTTTCAAGACGG). Positive clones were further identified by amplification and sequencing of 16S rRNA genes with universal primers 27F and 1492R (GenBank: OM956292, OM956294).

*A. muciniphila* strains AKK1 and AKK2 were inoculated in a synthetic liquid medium and incubated for 72 h at 37 °C under anaerobic conditions. At the stationary phase, the bacteria suspension was transferred to 20 mL of fresh synthetic liquid medium at a ratio of 1% for the physiological studies presented here. The inoculated broth was incubated as described above, and 1 mL samples were taken at 12 h intervals for 120 h. The samples were serially diluted 10-fold in PBS and 100 µL from each dilution was plated on LB agar plates in triplicate followed by incubation at 37 °C for 72 h. The bacterial colonies were counted and expressed as colony-forming units (CFUs)/mL.

The two strains were inoculated in synthetic the liquid medium at pH 2.0, 3.0, 4.0, 5.0, and 7.0 under anaerobic condition (85% N_2_, 10% CO_2_ and 5% H_2_) for 2 h [28]. Then the suspensions were serially diluted, plated onto BHI agar medium, and incubated at 37 °C for 72 h to count CFUs. The bile salt tolerance test was performed as described previously, but the synthetic liquid medium contained 0.1, 0.2, 0.3 and 0.4% bile salt [29].

On the other hand, two strains were inoculated in a synthetic liquid medium at 37 °C under anaerobic conditions for 48 h. The cultures were centrifuged at 4500× *g* for 20 min; the pellets were partly pasteurized at 70 °C for 30 min; and the bacteria were stored at −80 °C in 25% glycerol until needed.

### 3.2. Animal Experiments

All animal studies complied with the guidelines for the care and use of laboratory animals as described by the U.S. National Institutes of Health and were approved by the Animal Welfare and Research Ethics Committee at Jilin University (No. 20150601).

### 3.3. Mouse Trial

IFI204-deficient male mice were purchased from Nanjing Biomedical Research Institute of Nanjing University (Nanjing, China) and were subsequently backcrossed onto the C57BL/6J background at least eight generations. Six-week-old male C57BL/6J wild-type (WT) mice were purchased from Huafukang Biotechnology Co., Ltd. (Beijing, China) and housed in a 12 h light/dark cycle under controlled temperature and humidity. The normal diet (ND) contained 19% protein, 10% fat and 71% carbohydrate: total calories, 3.6 kcal/g. Liaoning Changsheng Biotechnology Co., Ltd., Benxi, China). The high-fat diet (HFD) contained 19.4% protein, 20.6% carbohydrate and 60% fat: total calories, 5.0 kcal/g (TP23300, Trophic Animal Feed High-Tech Co., Ltd., Nantong, China).

Twenty-four C57BL/6J mice were divided randomly into normal (ND, *n* = 8), high-fat diet groups (HFD, *n* = 8), and *A. muciniphila* AKK2-treated high-fat diet groups (HFD + AKK2, *n* = 8). The HFD + AKK2 group was challenged with 2 × 10^8^ CFU of the pasteurized *A. muciniphila* AKK2 strain orally in 150 µL of sterile PBS, and the ND and HFD groups were administered an equivalent volume of sterile PBS orally. The treatments with the above-mentioned regimens were performed daily. The body weights and fasting blood glucose of the mice were measured weekly following a 12 h fast. Blood samples were collected and placed in tubes at 4 °C for 2 h. The serum samples were then separated by centrifugation for 15 min at 2000× *g*, and stored at −20 °C until analysis.

### 3.4. Dog Trial

Male beagles about 1.5 years old were purchased from Kangping Experimental Animal Research Institute (Shenyang, China). All dogs were housed in individual cages and fed twice a day. All diets were prepared by our laboratory, and the content of protein and fat was tested to ensure essential daily nutrients. The HFD consisted of 63.16% cornmeal, 31.58% lard, 5.255% soy flour and 0.005% vitamins (8.78% protein, 19.56% fat, 71.66% carbohydrate). The normal diet (ND) consisted of 66.67% cornmeal, 22.22% lard, 11.105% soy flour and 0.005% vitamins (8.06% protein, 14.64% fat, 77.30% carbohydrate).

A total of 24 adult beagles were randomly divided into ND, HFD and HFD + AKK2 groups of eight each. The HFD + AKK2 group was challenged with 2 × 10^9^ CFU of pasteurized *A. muciniphila* AKK2 strain orally in 100 µL of sterile PBS, while the ND and HFD groups were orally administered an equivalent volume of sterile PBS. The body weight of the beagles was measured weekly following a 12 h fast. The intervention lasted for 9 weeks. At weeks 0, 2, 4, 6, 8 and 10, fresh fecal samples were collected from each dog for further microbiological analysis. Blood samples were collected from the forelimb veins prior to the commencement of experiments and at week 9. Serum was then separated from clotted blood by centrifugation for 15 min at 2000× *g*, and stored at −20 °C until needed for analysis.

### 3.5. Analyses of the Serum Biochemical Parameters

Serum lipids (total cholesterol, triglycerides, LDL cholesterol, HDL cholesterol) were measured by enzymatic photometric methods (Roche Diagnostics, Mannheim, Germany). The serum concentrations of alanine aminotransferase (ALT), aspartate aminotransferase (AST) and glutamine transpeptidase (GGT) were tested by commercial assay kits (Beckman Coulter, Brea, CA, USA).

### 3.6. Body Condition Scoring (BCS)

The visual method for the body condition score (BCS) was confirmed to be a semi-quantitative tool for assessing body fat composition in multiple animal health conditions. In this study, the body condition of all the beagles was determined using the nine-scale BCS system. The score was evaluated once a week for 9 weeks and performed according to the descriptions and illustrations provided by WSAVA [30]. Each dog was assessed by at least two persons who learned the BCS evaluation technique from a veterinarian.

### 3.7. Oral Glucose Tolerance Test (OGTT) and Insulin Tolerance Test (ITT)

At week 9 of the experimental schedule, all beagles underwent an OGTT following an overnight (18 h) fast as described previously [31]. The ITT was performed by commercial assay kits (Changjin Bioscience Co., Changsha, China).

### 3.8. Real-Time Quantitative Polymerase Chain Reaction (RT-qPCR)

The abundance of *A. muciniphila* was analyzed by RT-qPCR on a Light Cycler 96 platform (Roche, Mannheim, Germany). The DNA of fecal bacteria was extracted by TIANamp Stool DNA Kit (Tiangen, Beijing, China) according to the manufacturer’s instructions. The primer sequences used are listed in Table 1.

### 3.9. High-Throughput Sequencing of Intestinal Flora

The fecal samples of beagles were analyzed to determine the gut microbial composition. DNA was extracted and purified from each sample as previously described, and the V4–V5 region of the 16S rRNA gene was amplified using modified universal bacterial primers. Purified PCR products were sequenced using the HiSeq Illumina platform. The sequences were analyzed using the QIIME software [32]. The UPARSE pipeline was used to cluster the sequences with 97% similarity into operational taxonomic units (OTUs). An operating taxon with domain values of 0.99 was considered to be a genus. A representative sequence was selected for each OUT and was compared using an RDP classifier (v 2.2) [33].

### 3.10. Microcapsule Fabrication of A. muciniphila

The microcapsule of *A. muciniphila* AKK2 was prepared by a modified chitosan linked alginate method after being resuspended in a sterile alginate solution containing 5% trehalose and 5% sucrose and transferred into sterile mixed colloidal solution (*v*:*v* = 1:1). The mixed colloidal solution consisted of 2% low methoxy pectin and 1.5% sodium alginate and was autoclaved at 121 °C for 20 min. The mixture was then squeezed into a 0.3 M calcium chloride solution using a 1 mL injector to crosslink with calcium and form droplets. The crosslinked droplets were generated after shaking at 200 rpm for 30 min on a magnetic stirrer and then filtered and washed with sterile water, as previous research reported [34,35,36]. To improve formation efficiency, the microcapsules were then placed in a 10 mL microfiltered chitosan solution (0.4% *w*/*v* in 0.1 M acetic acid, adjusted to pH 6.0 with 1 M NaOH) to allow for coating the alginate droplet. Droplets were collected by filtration before washing and freeze-drying. The microcapsule formation efficiency was measured by the ratio of droplet bacteria to original bacteria.

### 3.11. Simulated Gastrointestinal Fluid Assay

Simulated gastric fluid consisted of 1% *w*/*v* pepsin and adjusted to pH 2.0 with 1 M HCl. Simulated intestinal fluid was made by mixing 5.3 mL monosodium phosphate (NaH_2_PO_4_) solution (0.2 mol/L) with 94.7 mL disodium phosphate (Na_2_HPO_4_) solution (0.2 mol/L) adjusted to pH 7.2 with 1 M HCl. The release of cells from the microcapsules was determined by placing 0.1 g microcapsules in 10 mL simulated gastric juice (pH 2.0) for 1 h, followed by 10 mL simulated intestinal juice (pH 7.2) for 2 h under 37 °C anaerobic conditions. During this process, aliquots (0.1 mL) were taken from the mixture, serially diluted, and plated onto BHI agar plates for colony enumeration. The lyophilized powder of nonencapsulated AKK2 was used as a control, and the same treatment as the previous microcapsules was performed. Three replicates of each experiment were performed independently.

### 3.12. Stability of Microcapsules

The stability of microcapsule was studied after 0, 7, 14 and 28 d of storage at 4 °C or 25 °C under anaerobic (85% N_2_, 10% CO_2_, 5% H_2_) or aerobic conditions. The bacteria released from the microcapsule were enumerated after treatment with a 3% sodium citrate solution. The lyophilized powder of nonencapsulated bacteria was used as a control, and the same treatment as the previous microcapsules was performed.

### 3.13. Statistical Analyses

Data were presented as mean ± SEM. All statistical analyses were performed using GraphPad Prism 7.0 (GraphPad, San Diego, CA, USA). Statistical analysis was performed by one-way ANOVA with the Tukey–Kramer post hoc test and Student’s *t*-test. A *p* value of < 0.05 was considered significant, and *p* < 0.01 was considered highly statistically significant (*, *p* < 0.05; **, *p* < 0.01; ***, *p* < 0.001).

## 4. Discussion

The prevalence of overweight/obesity has reached epidemic proportions. Compared with the classic mice or pig models, recent studies have reported the dog microbiome to be closer to that of a human [37]. Thus, we first verified the anti-obesity function of *A. muciniphila* AKK2, isolated from HFD-fed mice and observed its role in obese beagles. Results demonstrated the *A. muciniphila* AKK2 treatment, tended to reduce HFD-induced obesity in both, which was manifested in significantly lower levels of weight gain, fasting blood glucose, serum TC, and LDL-C. The above results indicated that *A. muciniphila* AKK2 is important for protection against HFD-induced obesity. 

A great number of studies have revealed that *A. muciniphila* and *Verrucomicrobia* are very rare or not detected in the digestive tract of dogs [38]. Some reasons might be inadequate sequence depth, the primers, 16S rRNA probes, or the reference sequence database [39,40]. This was also demonstrated by the very low relative abundance of *A. muciniphila* in fecal samples from healthy and obese beagles in our study.

The gold measurement standards for diagnosing of type II diabetes mellitus are fasting blood glucose and the oral glucose tolerance test (OGTT). In this study, the concentration of blood glucose was significantly higher in the HFD-induced obese beagles than for those in the normal group but still within normal range. Therefore, we speculated that obesity and type II diabetes in beagles may not necessarily be connected. Nevertheless, these parameters decreased significantly after the AKK2 treatment, which further confirmed that it is important for regulating blood glucose.

Many studies have found that HFD intake induced obesity and varying degrees of liver damage. In this study, along with the increase in body weight of beagles, the biomarkers of liver function (GGT, ALT, and AST) were also higher in the HFD group than in the normal group. However, AKK2 reduced these parameters in the HFD group, indicating the hepatoprotective activity of the *A. muciniphila* AKK2 in obese beagles.

Numerous studies have demonstrated that obesity is closely associated with the gut microbiota, which directly affects food digestion, absorption, and metabolism. Dietary patterns and habits play an important role in the regulation of the intestinal flora. Accumulated evidence reveals that probiotics significantly alleviate obesity-related dysbacteriosis [41]. In this study, fecal bacterial community sequencing showed that a high-fat diet alters gut microbiota composition and *A. muciniphila* supplementation increased the *Firmicutes/Bacteroidetes* (F/B) ratio in beagles. Furthermore, we found that obesity phenotypes such as weight gain and blood glucose concentration were inversely associated with the F/B ratio. At the phylum level, *A. muciniphila* reversed the decreased abundance of F*irmicutes* and *Fusobacteria* and the increased abundance of *Actinobacteria, Proteobacteria*, and *Bacteroidetes* in obese beagles. At the genus level, *A. muciniphila* increased the abundance of *Bifidobacterium* and *Lactobacillus* although the difference was not significant. Lu et al. observed that HFD feeding markedly decreased the abundance of *Lactobacillus reuteri*, *Lactobacillus mucosa*, *Lactobacillus johnsonii*, *Prevotella copri*, *and Clostridium perfringens* [42]. Therefore, the weight loss function of *A. muciniphila* may be related to regulating the abundance of these bacteria.

*A. muciniphila* supplemented orally is confronted with multiple impediments owing to the complex digestive environment, such as variable pH and high bile salt content in the small intestine. Besides, *A. muciniphila* is highly sensitive to oxygen, which limits its further application. Thus, it is essential that *A. muciniphila* be properly protected in the development of any functional probiotic products.

The diverse preparation of microcapsules can protect probiotics against harsh environments and improve their fluidity to provide convenient conditions for later product development. Alginate is a biocompatible polymer, commonly used in the formation of hydrogels, microspheres, fibres, and microcapsules. Chitosan-linked alginate hydrogels could form a polyelectrolyte complex (PEC) to reduce particle porosity [43,44,45]. Moreover, chitosan buffers excess protons in the gastric environment. In this study, we found that the abundance of *A. muciniphila* in the intestine of beagles was extremely low. The bile acids produced by the duodenum had a degrading effect, which significantly reduced the number of viable bacteria, making it impossible to maintain continuous colonization. However, after AKK2 was encapsulated by chitosan and alginate its survival improved in simulated gastric fluid and the intestinal environment, and it became more stable under different storage conditions. Thus, we hypothesize that the administration of *A. muciniphila* microcapsules was more conducive to the colonization of live bacteria in the gastrointestinal tract of animals.

In conclusion, this study revealed the beneficial role of anti-obesity treatments in beagles. *A. muciniphila* AKK2 promoted weight loss and ameliorated obesity-related metabolism in HFD-fed beagles. Moreover, *A. muciniphila* AKK2 restored HFD-induced changes in canine gut microbiota and promoted the abundance of probiotics, especially *Bifidobacterium* and *Lactobacillus.* These observations revealed that the anti-obesity effect of *A. muciniphila* in obese beagles was related to the regulation of intestinal flora. A better understanding of *A. muciniphila* may provide a new strategy for the effective treatment of overweight dogs.

## Figures and Tables

**Table 1:** The primer sequences for RT-PCR.

Primers	Nucleotide Sequence of Primers (5′-3′)
*A. muciniphila*-F	AGAGGTCTCAAGCGTTGTTCGGAA
*A. muciniphila*-R	GATGAGGTGGCAGACAGGAGAC
EUB-F	AGAGTTTGATCCTGGCTC
EUB-R	TGCTGCCTCCCGTAGGAGT

EUB-F and EUB-R, the bacterial universal primer set Eubac 27F and 338R, were reference genes.

**Table 2:** Survival rate of strains under different pH values (%).

Strains	2.0	3.0	4.0	5.0	7.0
AKK1	0.046	0.16	1.28	6.45	100
AKK2	0.075	0.20	2.76	10.6	100

AKK1, *A. muciniphila* AKK1, AKK2, *A. muciniphila* AKK2. The survival rate = the bacteria amount (various pH)/the bacteria amount (pH 7.0).

**Table 3:** The survival rate of strains under different bile salt concentration (%).

Strains	0.05	0.10	0.20	0.30	0.00
AKK1	54.9	23.2	9.6	1.08	100
AKK2	42.6	11.4	7.9	0.66	100

AKK1, *A. muciniphila* AKK1, AKK2, *A. muciniphila* AKK2. The survival rate = the bacteria amount (various bile salt concentration)/the bacteria amount (C _(bile salt)_ = 0.00).

**Table 4:** The body condition scoring results of beagles.

	ND		HFD		HFD + AKK2	
	Before	After	Before	After	Before	After
BCS	4.44 ± 0.54 ^a^	4.43 ± 0.45 ^a^	4.63 ± 0.35 ^a^	8.95 ± 0.51 ^b^	4.59 ± 0.30 ^a^	4.53 ± 0.34 ^a^

^a^ no significant difference; ^b^ Significant difference. versus Before.

**Table 5:** The microcapsule embedding number of bacteria log (CFU/mL).

Item	AKK2 Strain	Embedding Rate (%)
Number of viable bacteria before embedding	9.30 ± 0.01	81.80
Number of viable bacteria in filtrate and washing liquid	8.56 ± 0.03	-

**Table 6:** The releasing test in simulated gastric-intestinal fluid lg (CFU/g).

	AKK2 Microcapsule	AKK2 Strain
Original bacteria concentration	9.38 ± 0.02 ^a^	9.40 ± 0.01 ^a^
Simulated gastric fluid	8.76 ± 0.10 ^a^	6.05 ± 0.05 ^b^
Simulated intestinal fluid	7.39 ± 0.05 ^a^	4.89 ± 0.06 ^b^

Values are presented as the mean ± SEM. ^a^ no obviously difference. ^b^ Significant difference.

## Data Availability

The datasets used and/or analyzed during the current study are available from the corresponding author on reasonable request. The 16S rRNA genes of *Akkermansia muciniphila* strain AKK1 and AKK2 were deposited in GenBank under the accession numbers OM956292-OM956294.

## Abbreviations

*A. muciniphila*: *Akkermansia muciniphila*; NAFLD: non-alcoholic fatty liver disease; BSAVA: British Small Animals Veterinary Association; WSAVA: World Small Animal Veterinary Association; IBD: inflammatory bowel diseases; HFD: high-fat diet; FG: fasting glucose; IFI204: interferon-inducible protein 204; CFU: colony-forming unit; rRNA: ribosomal RNA; PBS: phosphate buffered saline; PCR: polymerase chain reaction; BHI: brain heart infusion; LB: luria broth; LDL: low density lipoprotein; HDL: high density lipoprotein; AUC: area under the curve.
